# Altered neurotransmitter function in CO_2_-exposed stickleback (*Gasterosteus aculeatus*): a temperate model species for ocean acidification research

**DOI:** 10.1093/conphys/cov018

**Published:** 2015-04-28

**Authors:** Floriana Lai, Fredrik Jutfelt, Göran E Nilsson

**Affiliations:** af1 Section for Physiology and Cell Biology, Department of Biosciences, University of Oslo, Oslo 0316, Norway; af2 Department of Biological and Environmental Sciences, University of Gothenburg, Gothenburg 41390, Sweden

**Keywords:** γ-Aminobutyric acid, brain, global change, hypercapnia, lateralization, temperate fish

## Abstract

Studies on the consequences of ocean acidification for the marine ecosystem have revealed behavioural changes in coral reef fishes exposed to sustained near-future CO_2_ levels. The changes have been linked to altered function of GABAergic neurotransmitter systems, because the behavioural alterations can be reversed rapidly by treatment with the GABA_A_ receptor antagonist gabazine. Characterization of the molecular mechanisms involved would be greatly aided if these can be examined in a well-characterized model organism with a sequenced genome. It was recently shown that CO_2_-induced behavioural alterations are not confined to tropical species, but also affect the three-spined stickleback, although an involvement of the GABA_A_ receptor was not examined. Here, we show that loss of lateralization in the stickleback can be restored rapidly and completely by gabazine treatment. This points towards a worrying universality of disturbed GABA_A_ function after high-CO_2_ exposure in fishes from tropical to temperate marine habitats. Importantly, the stickleback is a model species with a sequenced and annotated genome, which greatly facilitates future studies on underlying molecular mechanisms.

## Teaser

Behavioural changes in the stickleback caused by near-future CO2 levels can be restored by treatment with gabazine, revealing an involvement of the GABAA receptor ion. Importantly, these effects can be conveniently studied in the stickleback, with a sequenced and annotated genome and exceptionally well-characterized behaviour and physiology

## Introduction

Burning of fossil fuel continues to increase the atmospheric CO_2_ level, widely affecting the ocean chemistry in a process commonly referred to as ocean acidification. According to the fifth assessment report from the Intergovernmental Panel on Climate Change in 2013, the atmospheric CO_2_ level may reach 800–1150 µatm within this century (Collins *et al*., 2013), and the resultant increase in oceanic CO_2_ levels is now recognized as a serious threat to the marine ecosystem.

Initial experimental studies on the effects of these near-future CO_2_ levels on fish behaviour have focused on tropical species, suggested to be more sensitive to carbon chemistry changes owing to their high metabolic rate and therefore high CO_2_ exchange with water (Nilsson *et al*., 2012). These studies show that sustained exposure to such near-future CO_2_ levels causes an array of sensory and behavioural alterations in coral reef damselfishes. The alterations observed include reversed olfactory and auditory preferences (Munday *et al*., 2009; Dixson *et al*., 2010; Simpson *et al*., 2011), loss of behavioural lateralization (Domenici *et al*., 2012), loss of learning (Ferrari *et al*., 2012), increased boldness and activity (Munday *et al*., 2010) and reduced temporal resolution in the retinal response to light (Chung *et al*., 2014).

It has recently become clear that the behaviour of temperate fish also may be affected by elevated CO_2_. It was found that the three-spined stickleback (*Gasterosteus aculeatus*) displays decreased behavioural lateralization, learning, boldness and curiosity when exposed to 990 µatm CO_2_ (Jutfelt *et al*., 2013). Moreover, the Californian rockfish (*Sebastes diploproa*) shows increased anxiety when exposed to 1125 µatm CO_2_ (Hamilton *et al*., 2014).

For the coral reef fish, the neural mechanisms causing behavioural abnormalities have been found to involve altered function of the γ-aminobutyric acid (GABA) neurotransmitter, because normal behaviour can be restored effectively by a moderate dose of the specific GABA_A_-receptor antagonist gabazine (SR-95531; Nilsson *et al*., 2012; Chivers *et al*., 2014; Chung *et al*., 2014). Furthermore, Hamilton *et al.* (2014) suggested that the increase of anxiety in the Californian rockfish is linked to altered function of the GABA_A_ receptor.

γ-Aminobutyric acid is the major inhibitory neurotransmitter in vertebrate brains. The GABA_A_ receptor is an ion channel with conductance for Cl^−^ and HCO_3_^−^ (Bormann *et al*., 1987). A net inflow of these negatively charged ions into the postsynaptic neuron will cause hyperpolarization of the neuronal membrane that counteracts depolarizing excitatory input. When exposed to elevated CO_2_, fish regulate their acid–base balance to avoid acidosis by accumulating HCO_3_^−^, accompanied by a release of H^+^ and Cl^−^ to the water (Ishimatsu *et al*., 2008; Brauner and Baker, 2009). It is likely that such ion-regulatory mechanisms lead to altered transmembrane Cl^−^ and HCO_3_^−^ gradients in the brain during high-CO_2_ exposure, which in turn cause a reversal of GABA_A_-receptor function (Nilsson *et al*., 2012). It is also likely that the ion-regulatory changes and/or changes in GABA_A_-receptor function involve complex long-term perturbations, such as altered gene expression, because the behavioural dysfunctions are manifested only after several days of high-CO_2_ exposure and persist for several days after normal CO_2_ levels have been restored (Munday *et al.*, 2012).

Our ability to characterize the physiological and molecular mechanisms linking elevated CO_2_ to altered GABA_A_-receptor function would be greatly enhanced if studies can be done in a model species with a sequenced genome and well-characterized behaviour, life history and physiology. Clearly, the three-spined stickleback is such a species if it can be shown that the behavioural effects of high-CO_2_ displayed by this fish are linked to the GABA_A_ receptor. Consequently, the aim of this study was to test whether the loss of behavioural lateralization seen in high-CO_2_-exposed stickleback can be reversed by treatment with the specific GABA_A_-receptor antagonist, gabazine.

## Materials and methods

### Ethics statement

The experiment was conducted in accordance with Swedish law and regulations and approved by the Ethical Committee on Animal Experiments in Gothenburg, Sweden (ethical permit numbers 100-2010 and 151-2011).

### Experimental animals

Adult marine three-spined stickleback (*G. aculeatus*) were collected using a hand-trawl in the Gullmars Fjord (58° 15.781′ N, 11° 29.815′ E) on the Swedish west coast and kept at the nearby Sven Lovén Centre for Marine Sciences (Kristineberg), University of Gothenburg (Sweden). After being sedated using 2-phenoxyethanol in seawater (0.5 ml l^−1^), fish were tagged using subcutaneous implants of fluorescent elastomer tags (Northwest Marine Tech. Inc., Shaw Island, WA, USA), allowing us to keep track of individual variables measured during the experiment.

Fish were divided into eight groups and transferred to eight 80 l aquaria with either control or high-CO_2_ water. Initially, four fish were put into each aquarium. During experiments, the fish were kept with a 14 h–10 h light–dark cycle and fed twice a day *ad libitum* with frozen *Artemia* sp. nauplii (Kordon Golden Gate, Hayward, CA, USA). At the beginning of the experiment, fish weight was 1.30 ± 0.230 g and standard length 46.50 ± 2.219 mm (mean ± SD). Subsequent weight and length measures are given in Supplementary Figs S1 and S2.

### Manipulation of CO_2_

The eight aquaria (four for each treatment group) were continuously supplied with water from four header tanks, two providing aerated control water and two providing high-CO_2_ water. The header tanks were supplied with surface seawater pumped from the fjord. Water from the header tanks was gravity fed through silicone tubing to each holding aquarium at a rate of ∼1 l min^−1^. In the high-CO_2_ tanks, the level of CO_2_ was regulated by keeping the pH stable near 7.7 with a pH stat (Aqua Medic, Bissendorf, Germany) connected to a solenoid valve controlling the bubbling of the water with CO_2_. Temperature, pH and partial pressure of CO_2_ (pCO_2_) were measured daily from a randomly chosen aquarium from each exposure group. The pCO_2_ was measured with an infra-red CO_2_ probe connected to a submerged CO_2_-permeable membrane (GM70 CARBOCAP; Vaisala, Vantaa, Finland) equipped with an aspiration pump, as described by Munday *et al*. (2012). Alkalinity was measured every third day by taking a sample of 25 ml from one treatment and one control aquarium. These samples were filtered through a 2 µm filter and analysed in a titration machine (SI Analytics, Mainz, Germany) by stepwise addition of hydrogen chloride. Water salinity measures were provided by the field station's monitoring of surface water. The measured pCO_2_ in the treatment aquaria was 992.1 ± 119.3 μatm CO_2_ and the pH was 7.69 ± 0.057. The control aquaria had a pCO_2_ of 442.4 ± 71.0 μatm CO_2_ and a pH of 8.02 ± 0.052 (mean ± SD; Table 1).

**Table 1:** Water chemistry data measured daily (pCO_2_, salinity and temperature) and twice weekly (alkalinity and pH_tot_), during the 50 day exposure experiment

Parameter	Control	Elevated CO_2_
pCO_2_ (μatm)	442 ± 71	992 ± 119
Alkalinity [μmol (kg sea water)^−1^]	2162 ± 239	2149 ± 195
Salinity (practical salinity units)	25.9 ± 2.3	25.9 ± 2.3
Temperature (°C)	13.1 ± 2.4	13.1 ± 2.4
pH_tot_ (calculated)	8.02 ± 0.05	7.69 ± 0.06

Abbreviations: pCO_2_, partial pressure of carbon dioxide; pH_tot_, total pH. The pCO_2_, alkalinity, salinity and temperature were measured, while the total pH was calculated with CO_2_calc (USGS, St Petersburg, Florida, USA). The data are presented as means ± SD.

### Behavioural lateralization test

**Figure 1:**
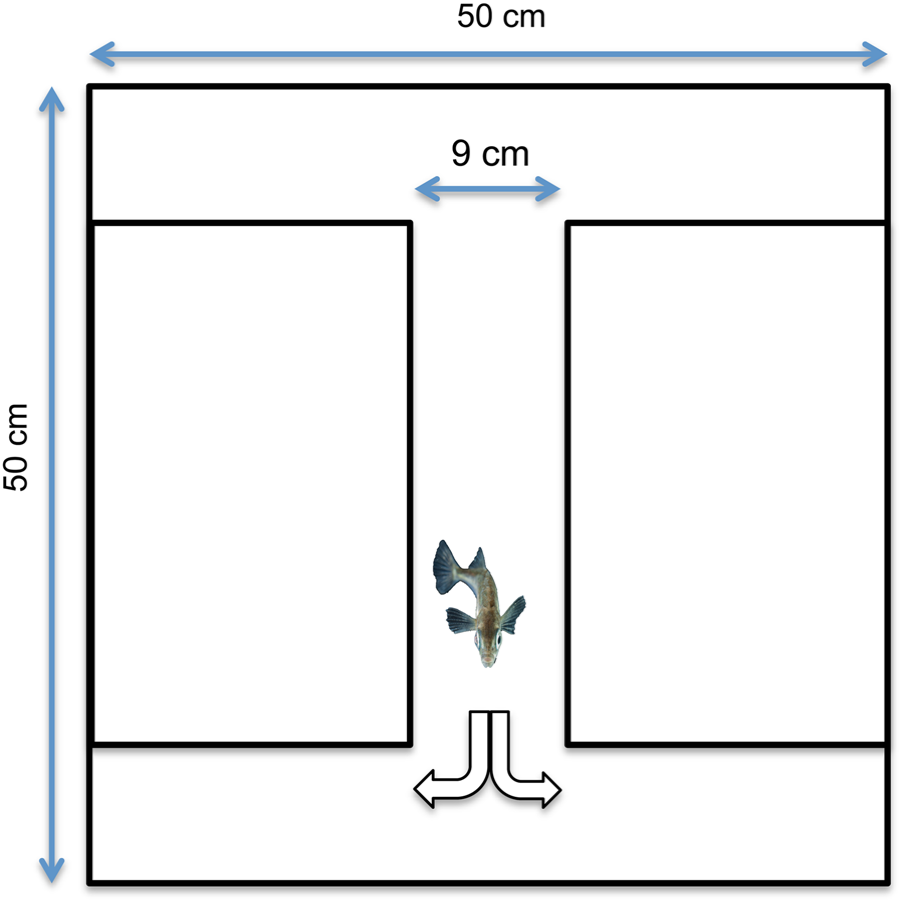
The double T-chamber used for the behavioural lateralization test. Each individual was encouraged to swim in the runway, and the preference on the left or right turn was recorded.

After 40 days of treatment, a double T-chamber was used to evaluate the effect of acidified water on behavioural asymmetry. The chamber measured 50 cm × 50 cm, with a runway 9 cm wide (Fig. 1) and was filled to high 5 cm of water supplied from the holding aquarium from which the fish was taken. Single individuals were introduced into the chamber and left for 3 min to explore the new environment. Subsequently, the fish was gently encouraged with a plastic rod to swim into the runway (unless it was already there) and then along the runway until the end, where the direction in which it turned was scored. It was then encouraged to swim back into the runway and, when reaching the opposing end, the direction of turn was again recorded. This procedure of making the fish swim back and forth in the runway was repeated until 20 turns were recorded. Care was taken to manoeuvre the fish as gently as possible, not touching it with the rod. As described by Jutfelt *et al*. (2013), the score of each individual's turn preference was used to calculate the relative and the absolute lateralization.

The behavioural lateralization was retested in the same individuals on day 50. This time, the fish were tested twice, once immediately before and once immediately after a 30 min treatment with gabazine. The treatment involved placing each fish in 200 ml jars containing 50 ml seawater with gabazine (4 mg l^−1^; Sigma-Aldrich Co., St Louis, MO, USA; Nilsson *et al*., 2012; Chung *et al*., 2014; Hamilton *et al*., 2014).

### Analysis

Length and weight were measured on days 0, 10, 20 and 50 and analysed using a two-way ANOVA followed by Sidak's multiple comparison *post hoc* test.

Lateralization indexes were calculated for each fish according to Domenici *et al*. (2012). In short, the mean for absolute lateralization indices (*L*_a_) reveals whether there is a significant lateralization at the population level regardless of direction of turn by individuals, and it is derived from the relative lateralization indices (*L*_r_) by removing any negative prefix (individuals with a majority of left turns yield negative *L*_r_ values).

The value of *L*_r_ was calculated as follows:

$$\left[\frac{\text{Turn to the right}-\text{Turn to the left}}{\text{Turn to the right}+\text{Turn to the left}}\right]\times 100$$

Differences in the absolute lateralization between control and high-CO_2_ fish on day 40 were analysed using Student's two-tailed *t*-test.

To analyse the effects of high CO_2_ and gabazine on day 50, two-way ANOVA with Sidak's multiple comparison test as the *post hoc* test was used. Values are given as means ± SEM if not otherwise stated.

## Results

**Figure 2:**
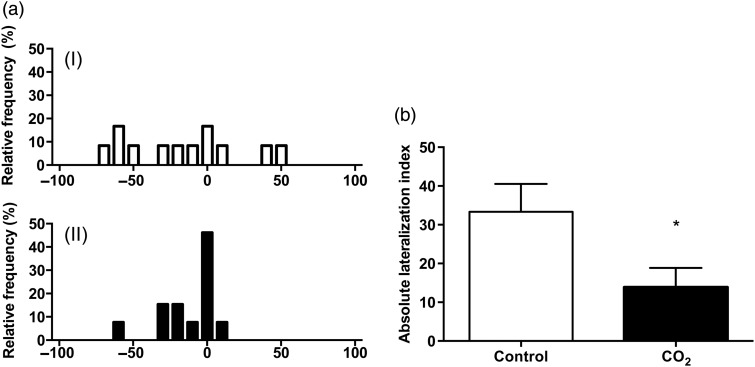
(**a**) Relative lateralization index (*L*_r_) of fish after 40 days of treatment. (I) *L*_r_ in control fish exposed to 442 μatm CO_2_; *n* = 12. (II) *L*_r_ in the CO_2_ group exposed to 992 μatm CO_2_ (*n* = 13). Comparison among groups was analysed with the *F*-test. (**b**) Absolute lateralization index after 40 days of treatment, revealing loss of lateralization after high-CO_2_ treatment. Carbon dioxide had significant effects on absolute lateralization (Student's two-tailed *t*-test, *P* = 0.034). Values are means ± SEM.

The three-spined stickleback kept in elevated CO_2_ demonstrated a complete loss of lateralization. Thus, after 40 days in the experimental aquaria, the high-CO_2_ treatment group had a significantly lower *L*_a_ than the control group (Fig. 2b). The *L*_a_ was 33.3 ± 7.2 in control fish (exposed to 442 μatm CO_2_) as a result of a significant individual preference for either turning left or right in the lateralization test (but with no population bias because the number of left and right turners were approximately equal). In contrast, fish exposed to 992 μatm CO_2_ for 40 days showed an *L*_a_ of 13.8 ± 5.0. Also, the *L*_r_ was differently distributed among fish exposed to control conditions and high CO_2_ (*F* = 4.15, *P* = 0.0213; Fig. 2a).

**Figure 3:**
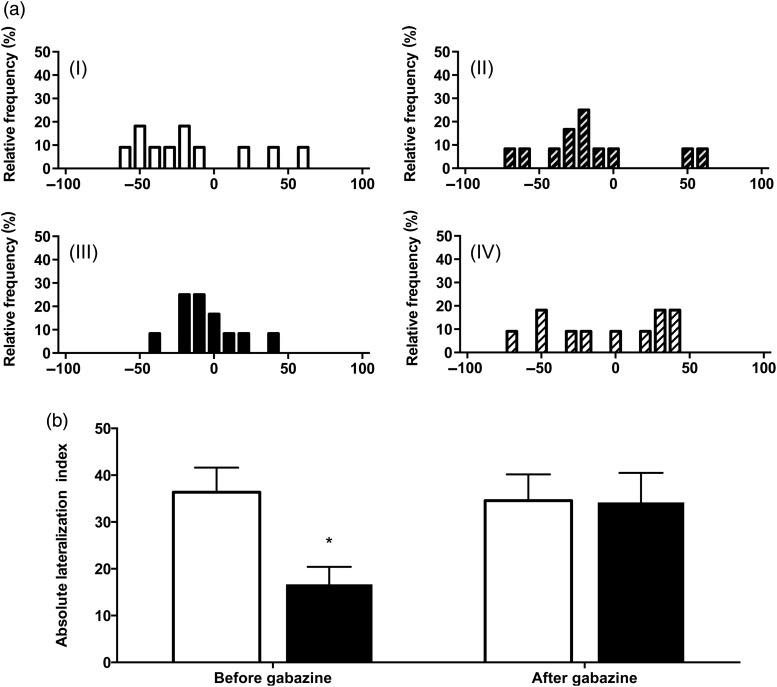
(**a**) Relative lateralization index of fish after 50 days of treatment. The values of *L*_r_ are shown for control fish exposed to 442 μatm CO_2_ before (I) and after (II) being kept in seawater containing gabazine (4 mg l^−1^) for 30 min (*n* = 11) and for fish exposed to 992 μatm CO_2_ before (III) and after (IV) being kept in seawater containing gabazine (4 mg l^−1^) for 30 min (*n* = 12). (**b**) The absolute lateralization index after 50 days of treatment in high CO_2_ was fully restored by GABA_A_-receptor antagonist treatment. The treatments had a significant effect (two-way ANOVA, *P* = 0.0269). *P < 0.05.

After 50 days in the experimental tanks, the fish were tested for lateralization again (Fig. 3), but this time in two subsequent runs, i.e. before and after treatment with gabazine (4 mg l^−1^ gabazine in seawater for 30 min). In the first run, control fish (*L*_a_ = 36.3 ± 5.3) and high-CO_2_ fish (*L*_a_ = 16.7 ± 3.7) showed virtually the same degree of lateralization as they did on day 40, with the suppressive effect of high-CO_2_ exposure on lateralization being retained (two-way ANOVA, *P* = 0.0269). In the second run, the lateralization in the high-CO_2_ group was completely restored by the gabazine treatment and, as a result, there was no difference between the control group treated with gabazine (*L*_a_ = 34.5 ± 5.6) and the high-CO_2_ group treated with gabazine (*L*_a_ = 34.2 ± 6.3; two-way ANOVA, *P* = 0.2229).

Lengths and weights over the experimental period did not differ between control fish and fish exposed to elevated CO_2_ (two-way ANOVA, *P* = 0.797 and 0.884, respectively; see Supplementary data). During the 50 day experiment, five control fish and four high-CO_2_-exposed fish died from unknown causes.

## Discussion

The result of the gabazine treatment shows that altered GABA_A_ receptor function is underlying the behavioural abnormalities displayed by stickleback after exposure to high-CO_2_ levels, exactly like previous findings in coral reef damselfish (Nilsson *et al*., 2012) and, recently, in rockfish (Hamilton *et al*., 2014).

Previous studies on coral reef damselfish (reviewed by Munday *et al*., 2012) have shown that the behavioural effects of high-CO_2_ treatment set in only after several days of sustained exposure and persist for several days after the high-CO_2_ exposure has ended, suggesting that alterations in gene expression may be involved. This could include changes in expression of genes for proteins involved in ion and pH regulation as well as the GABA_A_ receptor itself. Exposure to high-CO_2_ water has been found to reduce the response of the retina to fast stimuli, probably by altering the function of GABA_A_ receptors (Chung *et al*., 2014), and one may speculate that if other retinal functions are also altered this could contribute the loss of behavioural lateralization observed in this study as well as in previous studies on other species.

Most importantly, the present study suggests that the stickleback will provide us with a very useful model that opens up an avenue of experimental studies, including the examination of underlying molecular changes. For instance, transcriptomic studies will be aided by the annotated genome of this species, and we can also envision transgenerational studies, because this species can be bred readily in aquarium populations.

The three-spined stickleback is regarded as fairly tolerant of various environments, thriving in both freshwater and seawater (Pottinger *et al*., 2002; Östlund-Nilsson *et al*., 2006; Barrett *et al*., 2011), suggesting an extraordinary ion-regulatory plasticity. Nevertheless, its neural functions are apparently sensitive to sustained exposure to predicted future CO_2_ levels, as shown by the complete loss of behavioural lateralization in the present study, in the study by Näslund *et al*. (2015) and in other behavioural tests by Jutfelt *et al*. (2013).

Behavioural lateralization is thought to be a consequence of hemispheric specialization in the brain, allowing animals to carry out parallel processing and simultaneous responses to different stimuli (Bisazza *et al*., 1998; Dadda *et al*., 2012). In fish, the left optic lobe is connected to the right eye and vice versa, leading to a corresponding specialization of the eyes. If the right eye is, for example, focused on observing potential threats, pointing the head to the right and turning to the right allows the right eye to observe possible threats coming from behind. At the same time, the left eye can be focused on a different task, such as acquiring resources. Improved schooling performance, escape response, orientation and cognitive tasks are other suggested advantages of brain asymmetry (Bisazza and Dadda, 2005; Sovrano *et al*., 2005; Vallortigara and Rogers, 2005; Dadda *et al*., 2010). Loss of lateralization may therefore possibly affect fish fitness and survival.

Initially, it was hypothesized that highly active fish with a need for rapid oxygen uptake, and therefore large respiratory surface areas, would be more at risk for being affected by increases in the water CO_2_ level (Nilsson *et al*., 2012). The reason for this is that a large respiratory surface area should also lead to a fast release of CO_2_ from blood to water, approaching an equilibrium with the ambient water, making the blood carbonate system more sensitive to ambient changes in CO_2_. Indeed, fish in general have relatively low internal levels of CO_2_ in comparison to air-breathing animals owing to the high solubility of CO_2_ in water. Consequently, few would have regarded the stickleback as particularly vulnerable, because it is not a ‘high-performance’ species with a particularly high rate of gas exchange with the environment and because it is able to tolerate a wide range of environmental conditions. A worrying aspect of the present study is therefore that it indicates that altered GABA_A_-receptor function in response to sustained environmental hypercapnia may affect virtually any fish, at least in marine habitats.

At present, we do not know to what degree natural selection will be able to counteract neural effects of elevated CO_2_ in the relatively short period during which oceanic CO_2_ levels have been projected to reach close to 1000 µatm. What the present study shows is that individual acclimation is unlikely to be of help, because the behavioural change was in no way reduced after 50 days in the high-CO_2_ environment.

## Supplementary material

Supplementary material is available at *Conservation Physiology* online.

## Funding

This work was supported by the University of Oslo [to G.E.N. and F.L.], The Formas Research Council [to F.J.], The Swedish Research Council [to F.J. and G.E.N.] and the Royal Swedish Academy of Science [to F.L.].

## Author contributions

The study was conceived and designed by G.E.N. and F.J. Experiments and measurements were executed by F.L. All authors contributed to writing the paper.

## Supplementary Material

Supplementary Data
