# Supplementary material for: Altered neurotransmitter function in CO2-exposed stickleback (Gasterosteus aculeatus): a temperate model species for ocean acidification research
Source: Conserv Physiol. 2015 Apr 28;3(1):cov018. doi: 10.1093/conphys/cov018 (PMC4778464; doi:10.1093/conphys/cov018)
Supplement: Supplementary Data [file cov018supp.zip › cov018supp.docx]

**Supporting information**

**Supporting information legends**

**Supplementary Figure 1, 2.** **Fish´s length and weight during the exposures.** Measurements refer to day 0 n_control_ = 16, n_CO2_ = 16; day 10 n_control_ = 15, n_CO2_ = 15; day 20 n_control_ = 14, n_CO2_ = 13; day 50 n_control_ = 11, n_CO2_ = 12.

**Supplementary Figure 1**

**Supplementary Figure 2**
